# SYNJ2BP is associated with mitochondrial abnormalities and skeletal muscle atrophy in experimental models

**DOI:** 10.3389/fcell.2026.1996512

**Published:** 2026-10-28

**Authors:** Jian Zhang, Yuxia Yang, Xiangji Meng, XiaoFei Wang, Jihang Dai, Pei Zhang, Jingcheng Wang, Wenyong Fei

**Affiliations:** 1 Department of Orthopedics, The Affiliated Hospital of Yangzhou University, Yangzhou University, Yangzhou, China; 2 Department of Orthopedics, Northern Jiangsu People’s Hospital Affiliated to Yangzhou University, Yangzhou, China

**Keywords:** calcium homeostasis, IP3R1/GRP75/VDAC1, mitochondrial dysfunction, skeletal muscle atrophy, SYNJ2BP

## Abstract

Skeletal muscle atrophy is associated with mitochondrial dysfunction, but the contribution of synaptojanin 2 binding protein (SYNJ2BP) remains unclear. Proteomic profiling of human supraspinatus muscle identified increased SYNJ2BP abundance in atrophied tissue, together with changes in mitochondrial and calcium-related protein sets. Increased SYNJ2BP abundance was also observed in mouse supraspinatus muscle after tendon transection. In C2C12 cells, SYNJ2BP overexpression was accompanied by altered myogenic and atrophy-associated proteins, abnormal mitochondrial ultrastructure, lower ATP content, and increased Rhod-2 fluorescence. VBIT-4 attenuated several of these changes but also reduced SYNJ2BP abundance, limiting interpretation as a selective downstream rescue. SYNJ2BP, IP3R1, and GRP75 were recovered in VDAC1 immunoprecipitates. Increased VDAC1 bait recovery and differences in input abundance preclude interpreting greater protein recovery as stronger relative association. In a separate mouse hindlimb immobilization model, local SYNJ2BP knockdown was associated with preserved mitochondrial morphology, less muscle wasting, and improved physical performance. These complementary observations implicate SYNJ2BP in muscle wasting and motivate investigation of IP3R1/GRP75/VDAC1-associated calcium regulation. They do not establish a shared causal mechanism across models. The GFP-containing expression vector also necessitates independent validation of the reactive oxygen species and membrane-potential measurements. Further work should establish physiological expression relevance, cell differentiation status, and the direction and specificity of calcium transfer.

## 1 Introduction

Skeletal muscle supports movement, energy metabolism, and whole-body protein homeostasis. Muscle atrophy develops when protein degradation exceeds protein synthesis, resulting in reduced muscle mass and fiber size and, frequently, impaired strength and physical performance (Bonaldo and Sandri, 2013; Wang et al., 2022; Yin et al., 2021). Disuse during prolonged bed rest, denervation, and tendon injury can contribute to muscle wasting (Murgia et al., 2023; Nozdrenko et al., 2022; Shirakawa et al., 2021). Age-related muscle loss also contributes to sarcopenia, although sarcopenia encompasses deficits in muscle strength and function as well as muscle quantity (Ryall et al., 2008). The mechanisms underlying muscle atrophy include altered protein turnover, inflammation, impaired regeneration, and mitochondrial dysfunction (Bullard et al., 2016; Chen et al., 2023; Ji et al., 2022; Sharma et al., 2020; Tan et al., 2025; Yin et al., 2021; Yue et al., 2025). Understanding how these processes interact is essential for identifying interventions that preserve muscle function. Muscle preservation has also been investigated in underweight patients after stroke and in experimental diabetes, while muscle function is associated with exercise tolerance in chronic heart failure (Harrington et al., 1997; Yang et al., 2023; Yoshimura et al., 2021). Ferroptosis has emerged as another potential contributor to muscle atrophy (Sun et al., 2024).

Mitochondria generate ATP through oxidative phosphorylation and participate in calcium signaling, redox regulation, and cell-death pathways (Ansari et al., 2018; Ebata et al., 2023; Gunter et al., 2004). These functions are particularly important in skeletal muscle, where energy demand varies substantially with contractile activity. Mitochondrial dysfunction can compromise ATP availability, increase oxidative stress, and alter signaling pathways that regulate muscle protein turnover. Evidence from experimental and clinical studies links these changes to muscle wasting during glucocorticoid exposure, inactivity, and aging (Hepple, 2014; Hyatt et al., 2019; Lei et al., 2024; Liu et al., 2016). However, the upstream regulators that connect mitochondrial dysfunction to specific forms of muscle atrophy remain incompletely defined.

Calcium is an intracellular messenger that links muscle excitation to contraction and helps coordinate mitochondrial metabolism with cellular energy demand. Physiological calcium uptake supports mitochondrial function, whereas excessive accumulation can disrupt bioenergetics and promote oxidative stress and cell injury (Gunter et al., 2004; Paupe and Prudent, 2018; Zhang et al., 2021). Calcium exchange at endoplasmic reticulum (ER)–mitochondria contact sites involves a protein complex comprising inositol 1,4,5-trisphosphate receptors, glucose-regulated protein 75 (GRP75), and voltage-dependent anion channel 1 (VDAC1). IP3R1 mediates calcium release from the ER, GRP75 helps couple IP3Rs to VDAC1, and VDAC1 permits calcium passage across the mitochondrial outer membrane; transfer into the mitochondrial matrix additionally requires inner-membrane transport mechanisms (Liu et al., 2019; Meng et al., 2023; Shoshan-Barmatz et al., 2017; Xu et al., 2024; Zhang et al., 2021). Studies in non-muscle systems have linked disruption of this machinery to mitochondrial calcium imbalance, oxidative damage, and apoptosis (Liu et al., 2019; Meng et al., 2023; Xu et al., 2024; Zhang et al., 2024). Whether its regulation contributes to skeletal muscle atrophy requires further investigation.

SYNJ2BP is a mitochondrial outer membrane protein implicated in protein recruitment and ER–mitochondria contacts (Hartmann et al., 2020; Pourshafie et al., 2022). Its contribution to skeletal muscle atrophy is unclear. We used human supraspinatus muscle for discovery and a mouse supraspinatus tendon transection model to assess whether similar expression changes occur after tendon injury. A separate hindlimb immobilization model was used to test the effects of local SYNJ2BP manipulation in disuse-related wasting. These models address related but distinct questions. C2C12 experiments examined mitochondrial and muscle-related molecular phenotypes, calcium-associated fluorescence, and recovery of candidate proteins in VDAC1 immunoprecipitates. Together, the experiments test whether SYNJ2BP is relevant across these settings, without assuming that the same causal pathway operates in every model.

## 2 Materials and methods

### 2.1 Human supraspinatus muscle samples

Human supraspinatus muscle samples were obtained from patients undergoing arthroscopic surgery for rotator cuff injury at Subei People’s Hospital. The study was approved by the hospital ethics committee (approval no. 2021ky-001), and patients provided consent before tissue collection. Preoperative shoulder magnetic resonance imaging (MRI) was used to classify samples as control or atrophic according to the ratio of supraspinatus muscle area to supraspinatus fossa area. During surgery, approximately 3 × 3 × 3 mm specimens were collected and immediately frozen in liquid nitrogen for subsequent analysis.

### 2.2 Proteomic profiling and enrichment analysis

Three control and three atrophic human supraspinatus muscle samples were selected on the basis of preoperative MRI findings. Samples were frozen in liquid nitrogen and transported on dry ice to the commercial service provider for proteomic analysis. Differentially abundant proteins were identified and subjected to Gene Ontology (GO) enrichment analysis using GOstats and clusterProfiler. Gene set enrichment analysis (GSEA) was used to investigate coordinated changes in protein sets. Enrichment was evaluated using the normalized enrichment score and false discovery rate where applicable.

### 2.3 Mouse models and local SYNJ2BP manipulation

The animal experiments were approved by the Yangzhou University Laboratory Animal Welfare and Ethics Committee (protocol no. 2021ky-001). Twelve male C57BL/6J mice aged 6–8 weeks were used to establish a supraspinatus tendon transection model. The left shoulder underwent tendon transection, and the right shoulder served as a contralateral sham control. Surgery was performed under anesthesia. After shaving and disinfecting the shoulder, a 1–1.5 cm incision was made to expose the supraspinatus tendon. The tendon was completely transected at its insertion on the greater tubercle of the humerus, with care taken to preserve adjacent nerves, vessels, and muscles. The muscle and skin layers were then closed. The contralateral shoulder underwent exposure and wound closure without tendon transection. Supraspinatus muscles were collected after euthanasia, and atrophy was evaluated by hematoxylin and eosin (H&E) staining. Samples for molecular analysis were rapidly frozen in liquid nitrogen.

Twenty-four male C57BL/6J mice aged 4 weeks were housed at the Yangzhou University Animal Experiment Center at 24 °C ± 2 °C and 30% relative humidity under a 12 h light/12 h dark cycle, with free access to standard chow and water. Mice were randomly assigned to four groups (n = 6 per group): Control, Atrophy, Atrophy + OE-SYNJ2BP, and Atrophy + SH-SYNJ2BP. Adeno-associated viral (AAV) vectors for SYNJ2BP overexpression or knockdown were purchased from Shanghai Jike Biotechnology Co., Ltd. and diluted to 1.0 × 10^11^ vector genomes/mL. The tibialis anterior and gastrocnemius muscles of the left hindlimb received local injections of saline or the corresponding AAV preparation. Control mice received saline and remained unrestricted. Atrophy mice received saline and underwent left hindlimb immobilization. The two intervention groups underwent the same immobilization procedure and received the SYNJ2BP-overexpression or SYNJ2BP-knockdown vector, respectively. The right hindlimb remained unrestricted.

Under anesthesia, the left hindlimb was immobilized using a custom plaster cast to induce atrophy of the gastrocnemius and tibialis anterior muscles. At the 6-week experimental endpoint, mice were evaluated and anesthetized with isoflurane using an R500 anesthesia system. The left hindlimb was shaved and disinfected with 70% alcohol. After skin incision and careful dissection, the gastrocnemius and tibialis anterior muscles were collected for subsequent analyses.

Body weight was recorded at baseline and weekly thereafter, giving seven measurements over 6 weeks. At the endpoint, the excised gastrocnemius and tibialis anterior muscles were weighed immediately before cryopreservation.

### 2.4 Histology and immunostaining

Human supraspinatus muscle samples were cryosectioned and subjected to heat treatment and endogenous peroxidase blocking before antibody incubation and chromogenic detection. Stained sections were examined by light microscopy. Staining intensity and distribution were analyzed using ImageJ.

Paraffin-embedded sections were deparaffinized in xylene and rehydrated through an alcohol series. Nuclei were stained with hematoxylin, and the cytoplasm was counterstained with eosin. Sections were then dehydrated and mounted. Images were acquired by light microscopy, and muscle fiber diameter was measured using Photoshop.

Immunofluorescence images of MYOD and MYOG were acquired in C2C12 cells (Figure 1B). SYNJ2BP and VDAC1 signals were examined alongside mitochondrial and nuclear labeling (Figure 2B).

**FIGURE 1:**
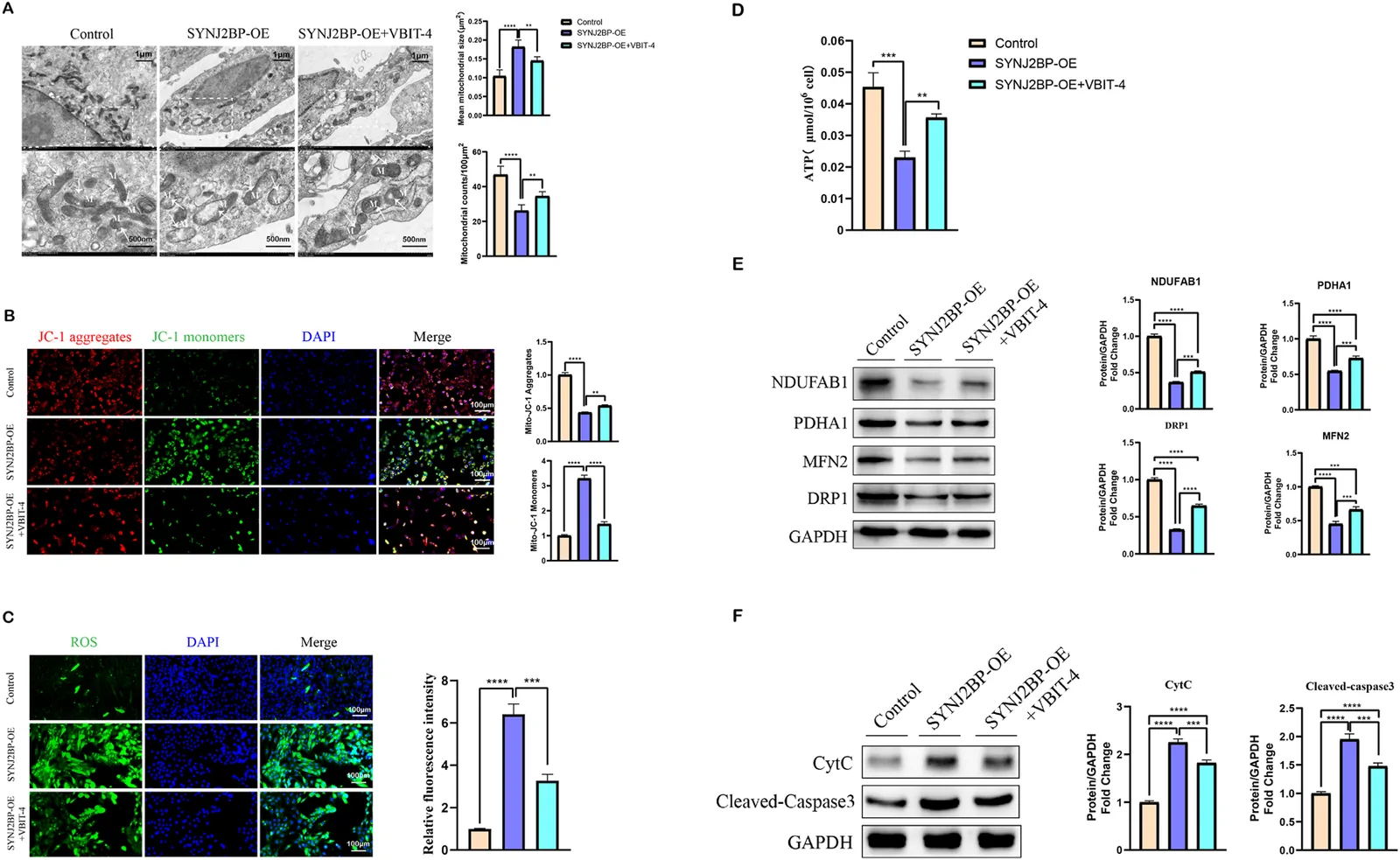
Supraspinatus muscle atrophy in humans and mice. Note. **(A)** Representative shoulder MRI and supraspinatus muscle-to-fossa area ratio in human control and atrophy groups. **(B)** Representative mouse supraspinatus muscles. **(C)** H&E-stained mouse supraspinatus sections and quantification of muscle fiber diameter. Scale bars: 100 μm in the upper row and 40 μm in the lower row. Data are presented as mean ± SD where quantified. *p < 0.05, **p < 0.01, ***p < 0.001, ****p < 0.0001; ns, not significant, where shown. Comparisons are indicated in the panels. The mouse shoulder comparison is paired within animals.

**FIGURE 2:**
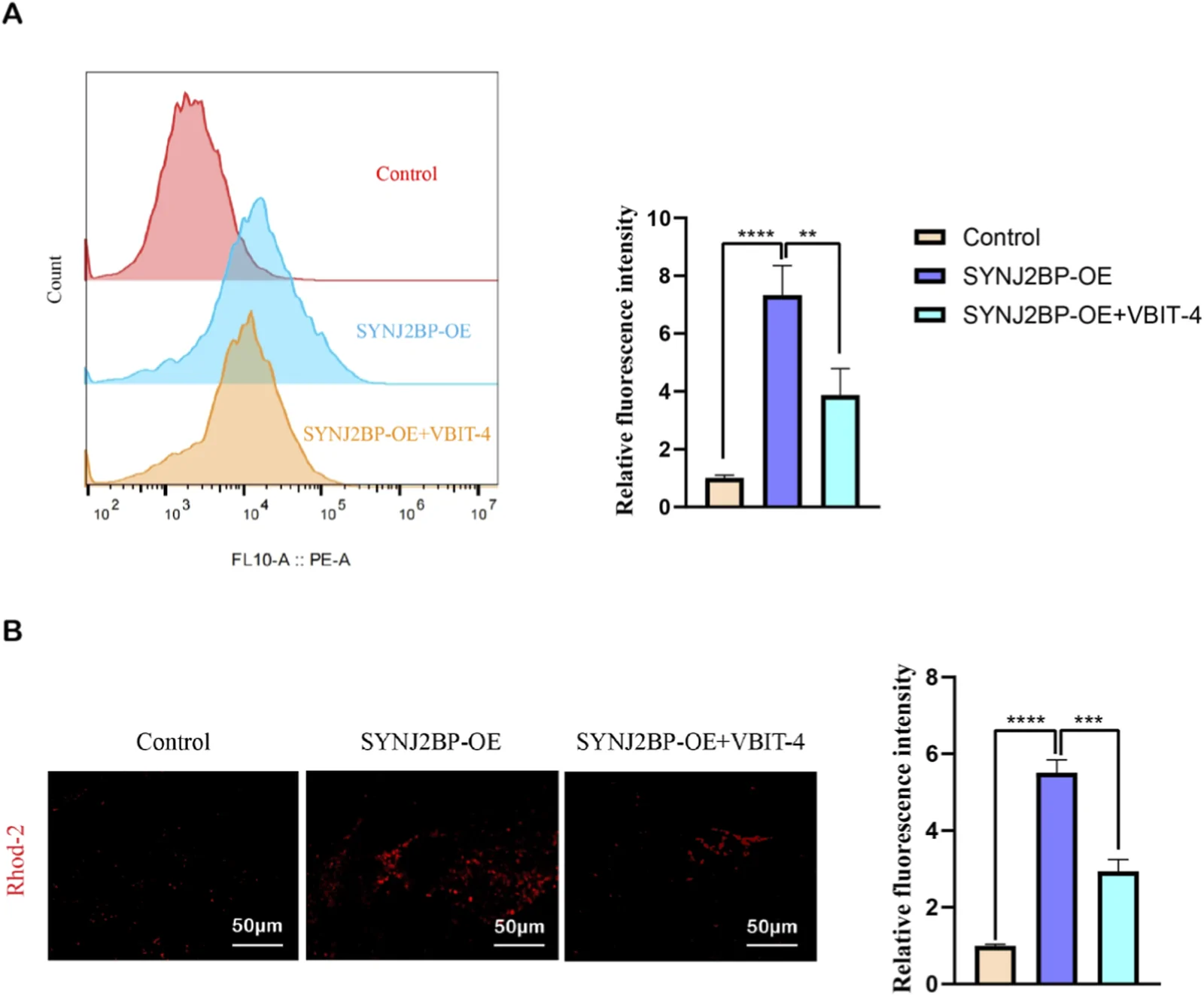
Increased SYNJ2BP abundance in atrophied supraspinatus muscle. Note. **(A)** Volcano plot of differentially abundant proteins in human supraspinatus muscle. **(B)** SYNJ2BP immunohistochemistry in human tissue. Scale bar: 20 μm. **(C,D)** Western blots and quantification of SYNJ2BP, MYOD, MYOG, and MYHC in human **(C)** and mouse **(D)** control and atrophic supraspinatus muscle. Data are presented as mean ± SD where quantified. *p < 0.05, **p < 0.01, ***p < 0.001, ****p < 0.0001; ns, not significant, where shown. Comparisons are indicated in the panels. The proteomic discovery cohort comprised three human samples per group.

### 2.5 C2C12 culture and experimental treatments

Mouse C2C12 myoblasts were purchased from Shanghai Baililai Biotechnology Co., Ltd. Cells were maintained in complete Dulbecco’s modified Eagle’s medium at 37 °C in a humidified atmosphere containing 5% CO_2_. The culture medium was replaced every 2–3 days, and cell growth was monitored regularly.

A SYNJ2BP-overexpressing lentivirus was generated using the GV492 vector, with the construct Ubi-MCS-3FLAG-CBh-gcGFP-IRES-puromycin. C2C12 cells were seeded and cultured overnight to reach approximately 30%–50% confluence. Lentivirus was thawed on ice and added at a multiplicity of infection of 10. The volume added was calculated as follows: virus volume (μL) = multiplicity of infection × cell number ÷ virus titer (transducing units/mL) × 1,000. Virus-containing medium was replaced the following day. Transduction was assessed by fluorescence microscopy on day 4 and confirmed by western blotting. The cellular experiments compared control, SYNJ2BP-OE, and SYNJ2BP-OE + VBIT-4 groups.

VBIT-4 was included to explore the involvement of VDAC-related processes because it was originally reported to interact with VDAC1 and inhibit oligomerization-associated apoptosis (Ben-Hail et al., 2016).

### 2.6 Western blotting and co-immunoprecipitation

Cells or tissues were lysed in RIPA buffer containing protease and phosphatase inhibitors. Lysates were clarified by centrifugation at low temperature, and protein concentrations were determined. Protein samples were mixed with loading buffer, heated at 95 °C–100 °C for 5–10 min, separated by gel electrophoresis, and transferred to membranes. After blocking, membranes were incubated with primary and secondary antibodies. Protein bands were detected using an enhanced chemiluminescence substrate and a chemiluminescence imaging system.

C2C12 cells (2–5 × 10^7^) were harvested for protein extraction. Clarified lysates were quantified using a BCA assay, and an aliquot was retained as the input control. Lysates were precleared with 20 μL of SweMagrose Protein G magnetic beads and centrifuged at 2,000 × g for 5 min at 4 °C. The supernatant was divided between an immunoprecipitation group and a species-matched normal IgG control. Antibodies were added at the manufacturer’s recommended dilution, and samples were incubated overnight at 4 °C. Protein G magnetic beads (100 μL) were then added, followed by incubation with gentle agitation for 2–4 h at 4 °C. Beads were collected magnetically and washed four times with 500 μL of prechilled Tris-buffered saline. Bound proteins were eluted in 100 μL of 1× reducing loading buffer, heated at 95 °C for 10 min, and centrifuged at 1,000 × g for 5 min at 4 °C. The supernatants were analyzed by western blotting.

Immunoblot signals were expressed relative to GAPDH or β-actin. For co-immunoprecipitation, the partner-to-VDAC1 recovery ratio was calculated by dividing each partner fold recovery by the VDAC1 fold recovery (Table 1). These descriptive ratios do not account for input protein abundance.

**TABLE 1:** Predicted SYNJ2BP–VDAC1 interface energy.

Receptor	Ligand	ΔiG (kcal/mol)
SYNJ2BP	VDAC1	−7.9

ΔiG is the reported interface solvation free-energy change from PDBePISA. It is not an experimentally measured binding free energy or dissociation constant.

### 2.7 Transmission electron microscopy

C2C12 cells were fixed, dehydrated, embedded, and polymerized for 2 days. Sections were stained with 2% uranyl acetate in ethanol and 2.6% lead citrate. Copper grids were dried overnight at room temperature before imaging by transmission electron microscopy. Mitochondrial profile size and profile counts were quantified using ImageJ. Muscle specimens were also examined to assess mitochondrial ultrastructure *in vivo*.

### 2.8 Fluorescence assays

Cellular reactive oxygen species (ROS) were assessed using DCFH-DA diluted in serum-free medium to a final concentration of 10 μmol/L.

The probe solution was added to cells under protection from light; 50 μL per well was used for 96-well plates. A nuclear stain was applied to label nuclei. Images were acquired by laser confocal microscopy, and fluorescence intensity was quantified using ImageJ.

Mitochondrial membrane potential was assessed using JC-1. The probe was dissolved in dimethyl sulfoxide according to the manufacturer’s instructions and diluted in serum-free medium to 5 μg/mL. Cells were treated with 0.5 mL of working solution, washed two or three times with JC-1 staining buffer, and imaged by laser confocal microscopy. Red aggregate and green monomer fluorescence were evaluated as indicators of mitochondrial membrane potential.

Calcium-associated fluorescence was assessed with Rhod-2 AM by flow cytometry and fluorescence microscopy (Figure 3).

**FIGURE 3:**
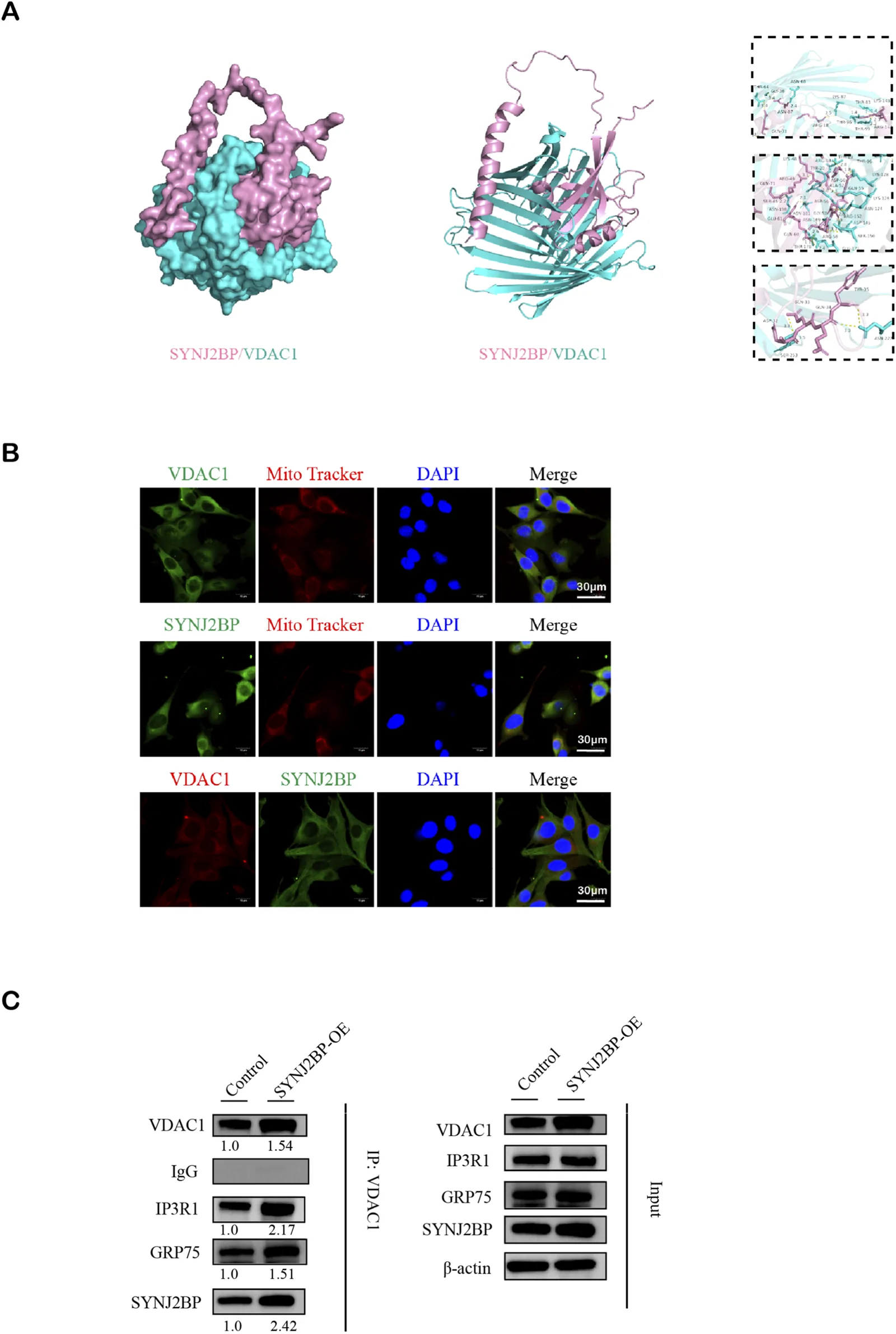
Mitochondrial and calcium related changes in atrophic muscle. Note. **(A)** GO enrichment of upregulated and downregulated protein sets in human supraspinatus muscle. **(B)** GSEA plot for calcium ion binding. **(C,D)** Western blots and quantification of IP3R1, GRP75, and VDAC1 in human **(C)** and mouse **(D)** supraspinatus muscle. Data are presented as mean ± SD where quantified. *p < 0.05, **p < 0.01, ***p < 0.001, ****p < 0.0001; ns, not significant, where shown. Comparisons are indicated in the panels. Proteomic discovery used three human samples per group. The mouse shoulder comparison is paired.

### 2.9 ATP content

ATP content was determined using an absorbance-based assay, with absorbance measured at 340 nm. ATP standards were included for quantification. Values in Figure 4D are expressed as μmol per 10^6^ cells.

**FIGURE 4:**
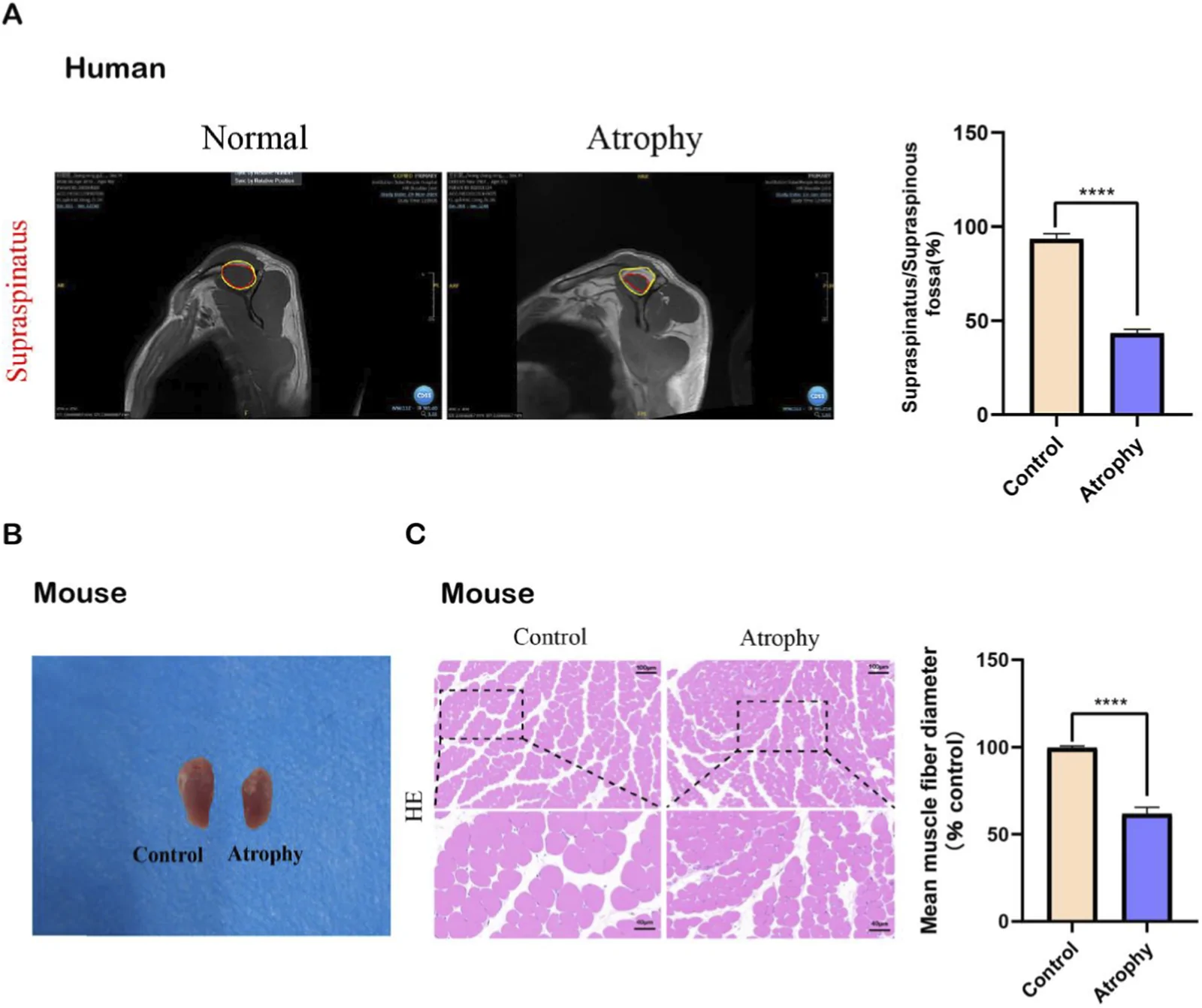
Effects of SYNJ2BP overexpression and VBIT-4 on muscle related markers. Note. C2C12 cells were compared across Control, SYNJ2BP-OE, and SYNJ2BP-OE + VBIT-4 groups. **(A)** Western blots and quantification of MYOD, MYOG, MYHC, Atrogin-1, and MuRF-1. **(B)** MYOD and MYOG immunofluorescence and quantification. Scale bar: 100 μm. **(C)** Western blots and quantification of SYNJ2BP, IP3R1, GRP75, and VDAC1. Data are presented as mean ± SD where quantified. *p < 0.05, **p < 0.01, ***p < 0.001, ****p < 0.0001; ns, not significant, where shown. Comparisons are indicated in the panels. Vector-encoded GFP may contribute to green-channel fluorescence. Panel C also shows reduced SYNJ2BP abundance with VBIT-4 treatment. These assays do not by themselves establish mature-myotube atrophy.

### 2.10 Protein docking

SYNJ2BP–VDAC1 docking was performed using GRAMM. Candidate complexes were evaluated using PDBePISA, and a selected conformation was visualized in PyMOL 2.3.0. The reported interface solvation free-energy change (ΔiG) was used to describe the predicted interface. Docking was treated as a structural hypothesis and interpreted alongside colocalization and co-immunoprecipitation data.

### 2.11 Mouse muscle function and exercise performance

Hindlimb grip strength was assessed using a TSE 303500-M/E-1 grip strength tester at the Yangzhou University Laboratory Animal Center. Each mouse was positioned with its hind paws on a grid connected to a force sensor and gently pulled backward until the left hind paw released the grid. The peak force was recorded, and each mouse underwent three trials.

Endurance was assessed using a TSE 303401-RATS treadmill at the Yangzhou University Laboratory Animal Center. The treadmill was set at a 10° incline and an initial speed of 10 m/min, with electrical stimulation used during testing. After 20 min, speed was increased by 1 m/min every 10 min until exhaustion. Exhaustion was identified by failure to maintain pace and sustained contact with the rear of the apparatus for up to 30 s, accompanied by rapid breathing and signs of fatigue. Maximum running time and distance were recorded.

### 2.12 Statistical analysis and experimental units

Quantitative data are presented as mean ± standard deviation. The human proteomic cohort comprised three samples per group. The tendon-transection experiment included 12 mice with contralateral sham controls. The immobilization experiment included 24 mice allocated to four groups of six. Body weight was measured at baseline and weekly for 6 weeks. Statistical significance is denoted by *p < 0.05, **p < 0.01, ***p < 0.001, and ****p < 0.0001; ns indicates not significant.

## 3 Results

### 3.1 Supraspinatus tendon injury is associated with muscle atrophy in humans and mice

Preoperative MRI showed a lower supraspinatus muscle-to-fossa area ratio in patients classified as having muscle atrophy than in the control group (Figure 1A). In mice, supraspinatus tendon transection produced a corresponding reduction in muscle size (Figure 1B). H&E staining showed a smaller muscle fiber diameter in the transected group than in the contralateral sham group (Figure 1C), supporting successful induction of supraspinatus muscle atrophy.

### 3.2 SYNJ2BP abundance increases in atrophied supraspinatus muscle

Proteomic profiling of three control and three atrophic human supraspinatus samples identified increased SYNJ2BP abundance in the atrophy group (Figure 2A). Immunohistochemistry and western blotting confirmed higher SYNJ2BP expression in atrophic human muscle, and a similar increase was observed in mouse supraspinatus muscle after tendon transection (Figures 2B–D). In both species, this increase was accompanied by lower expression of the myogenic proteins MYOD, MYOG, and MYHC (Figures 2C,D). These findings linked SYNJ2BP upregulation to muscle atrophy and motivated further investigation of its function.

### 3.3 Proteomic changes implicate mitochondrial processes and calcium binding

GO analysis of differentially abundant proteins identified downregulated protein sets related to mitochondrial organization, respiration, and oxidative phosphorylation in atrophic human muscle (Figure 3A). GSEA showed positive enrichment of the calcium ion binding gene set (Figure 3B). These findings suggested altered mitochondrial and calcium-related processes, although enrichment analysis alone does not establish mitochondrial dysfunction or channel activity. Consistent with altered ER–mitochondria calcium signaling, western blotting showed higher IP3R1, GRP75, and VDAC1 abundance in atrophic supraspinatus muscle from both humans and mice (Figures 3C,D).

### 3.4 VBIT-4 attenuates SYNJ2BP associated changes in myogenic and atrophy markers

To investigate the effects of SYNJ2BP, we compared control C2C12 cells, cells overexpressing SYNJ2BP, and SYNJ2BP-overexpressing cells treated with VBIT-4. SYNJ2BP overexpression was accompanied by lower MYOD, MYOG, and MYHC abundance and higher Atrogin-1 and MuRF-1 abundance (Figure 4A). MYOD and MYOG fluorescence showed corresponding changes (Figure 4B), although vector-encoded GFP may contribute to the green-channel signal. VBIT-4 partially attenuated the protein changes. It also reduced SYNJ2BP abundance relative to the overexpression group, alongside reductions in IP3R1, GRP75, and VDAC1 (Figure 4C). The treatment effect may therefore reflect a change in SYNJ2BP abundance as well as downstream or off-target drug effects. These observations describe a muscle-related molecular phenotype in C2C12 cells. Changes in these markers alone do not establish atrophy of mature myotubes.

### 3.5 SYNJ2BP overexpression is associated with mitochondrial abnormalities

Transmission electron microscopy showed disrupted cristae, vacuolization, enlarged mitochondrial profiles, and fewer mitochondrial profiles in SYNJ2BP-overexpressing C2C12 cells. These changes were attenuated in the VBIT-4-treated group (Figure 5A). JC-1 images showed lower red aggregate fluorescence and higher green-channel fluorescence after SYNJ2BP overexpression, while DCFH-DA images showed higher green-channel fluorescence (Figures 5B,C). Because the expression vector encodes gcGFP, these observations cannot establish decreased mitochondrial membrane potential or increased ROS without suitable fluorescence controls or independent validation. ATP content was lower after SYNJ2BP overexpression and higher with VBIT-4 treatment (Figure 5D). NDUFAB1, PDHA1, MFN2, and DRP1 abundance also decreased after overexpression and increased with VBIT-4 relative to the overexpression group (Figure 5E). Total cytochrome c and cleaved caspase-3 abundance increased after overexpression and decreased with treatment (Figure 5F). These results support structural and energetic abnormalities with changes in apoptosis-associated proteins. Total cytochrome c abundance does not demonstrate release into the cytosol.

**FIGURE 5:**
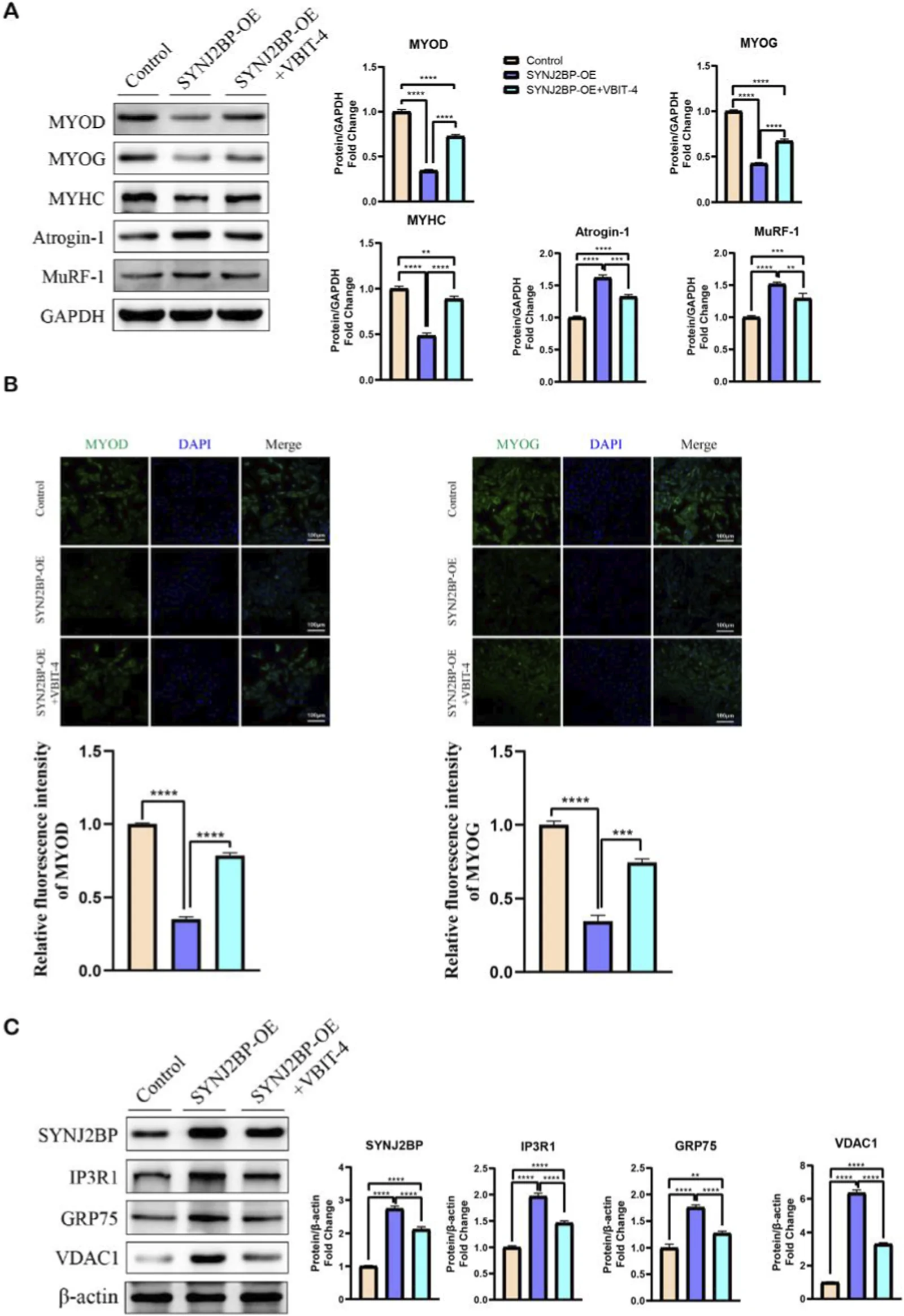
Mitochondrial abnormalities associated with SYNJ2BP overexpression. Note. Panels A–C. Control, SYNJ2BP-OE, and SYNJ2BP-OE + VBIT-4 C2C12 cells were compared. **(A)** Transmission electron micrographs and quantification of mitochondrial profile size and number. Scale bars are shown in the panels. **(B)** JC-1 red and green fluorescence. **(C)** DCFH-DA assay fluorescence. Scale bars in B and C 100 μm. Data are presented as mean ± SD where quantified. *p < 0.05, **p < 0.01, ***p < 0.001, ****p < 0.0001; ns, not significant, where shown. Comparisons are indicated in the panels. Vector-encoded GFP may contribute to green-channel signals in panels B and C. Note. Panels D–F. The same three groups are compared. **(D)** Cellular ATP content. **(E)** Western blots and quantification of NDUFAB1, PDHA1, MFN2, and DRP1. **(F)** Western blots and quantification of cytochrome c and cleaved caspase-3. Data are presented as mean ± SD where quantified. *p < 0.05, **p < 0.01, ***p < 0.001, ****p < 0.0001; ns, not significant, where shown. Comparisons are indicated in the panels. Cytochrome c denotes total abundance, not a measurement of cytosolic release.

### 3.6 SYNJ2BP overexpression increases calcium associated Rhod-2 fluorescence

Rhod-2 AM fluorescence was assessed by flow cytometry and fluorescence microscopy. SYNJ2BP-overexpressing cells showed higher fluorescence than control cells, whereas VBIT-4 treatment reduced the signal (Figures 6A,B). These findings indicate altered calcium-associated fluorescence. Rhod-2 fluorescence alone does not establish mitochondrial calcium overload or resolve the direction and rate of ER-to-mitochondria calcium transfer.

**FIGURE 6:**
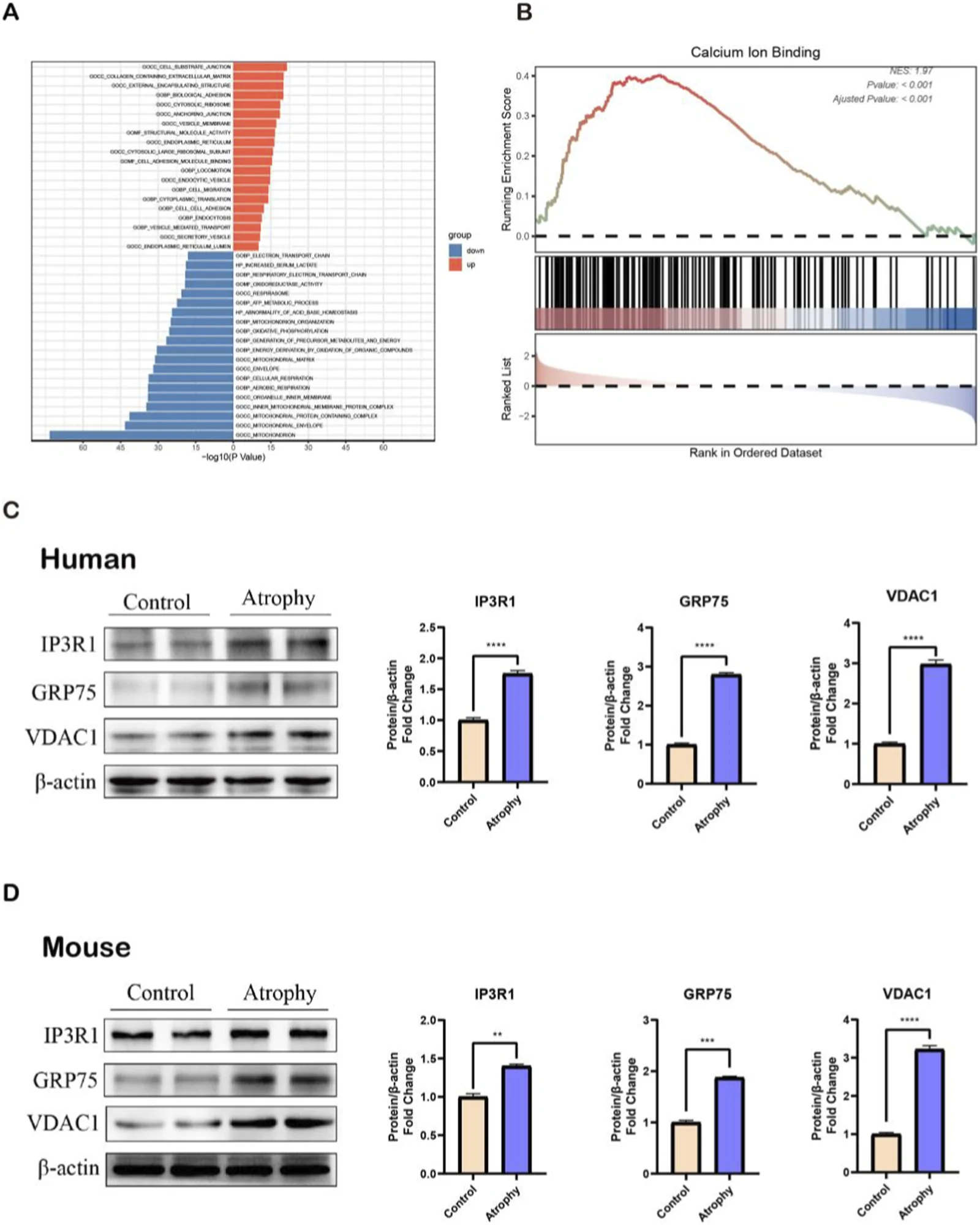
Increased Rhod-2 fluorescence following SYNJ2BP overexpression. Note. **(A)** Flow cytometry and fluorescence quantification in Control, SYNJ2BP-OE, and SYNJ2BP-OE + VBIT-4 C2C12 cells. **(B)** Rhod-2 fluorescence microscopy and quantification in the same groups. Scale bar: 50 μm. Data are presented as mean ± SD where quantified. *p < 0.05, **p < 0.01, ***p < 0.001, ****p < 0.0001; ns, not significant, where shown. Comparisons are indicated in the panels. Rhod-2 fluorescence is a calcium-associated readout and does not independently establish mitochondrial localization or directional calcium flux.

### 3.7 SYNJ2BP is recovered with VDAC1 in co-immunoprecipitation

Docking identified a candidate SYNJ2BP–VDAC1 interface with a ΔiG of −7.9 kcal/mol (Figure 7A; Table 1). This structural prediction does not establish binding affinity in cells. Fluorescence microscopy showed overlap between SYNJ2BP or VDAC1 signals and mitochondrial staining, as well as overlap between the two protein signals (Figure 7B). Vector-encoded GFP may contribute to the green-channel signal. In VDAC1 immunoprecipitates, fold recoveries relative to control were 1.54 for VDAC1, 2.17 for IP3R1, 1.51 for GRP75, and 2.42 for SYNJ2BP (Figure 7C). The approximate partner-to-VDAC1 recovery ratios were 1.41, 0.98, and 1.57 for IP3R1, GRP75, and SYNJ2BP, respectively (Table 2). GRP75 recovery relative to VDAC1 was therefore close to the control value. These descriptive ratios do not account for input protein abundance. The findings support co-recovery of these proteins but do not establish increased relative association, binding affinity, or complex stoichiometry.

**FIGURE 7:**
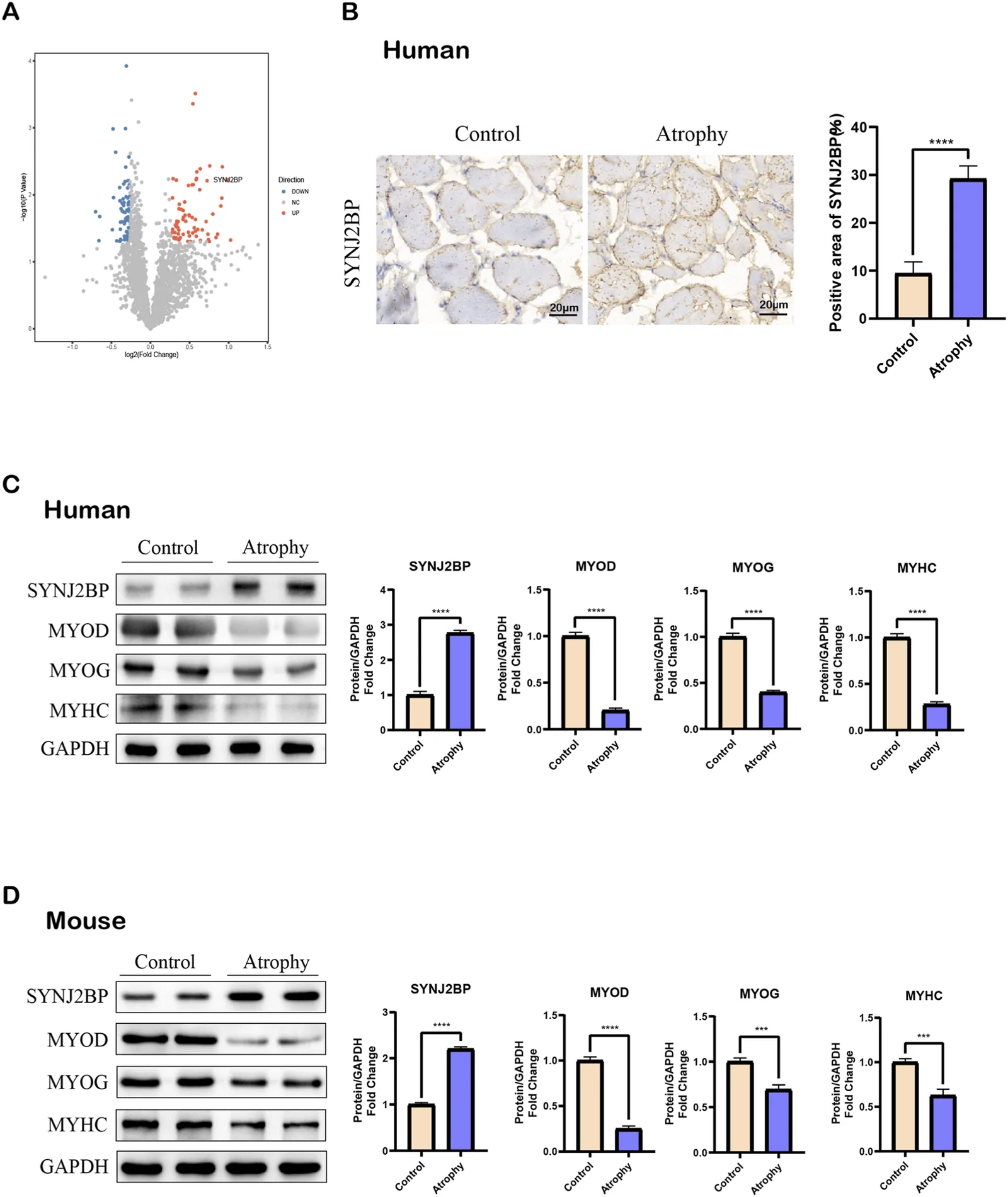
Association of SYNJ2BP with VDAC1. Note. **(A)** Predicted SYNJ2BP–VDAC1 docking interface. **(B)** Fluorescence images showing overlap between SYNJ2BP or VDAC1 signals and mitochondrial staining, and between SYNJ2BP and VDAC1 signals. Scale bar: 30 μm. **(C)** Co-immunoprecipitation of SYNJ2BP, IP3R1, and GRP75 with VDAC1 in control and SYNJ2BP-overexpressing cells. Values below the immunoprecipitation bands indicate fold recovery relative to control. Partner-to-VDAC1 recovery ratios are summarized in Table 1.

**TABLE 2:** Descriptive co-immunoprecipitation recovery relative to VDAC1.

Protein	Partner fold recovery	VDAC1 fold recovery	Partner-to-VDAC1 ratio
IP3R1	2.17	1.54	1.41
GRP75	1.51	1.54	0.98
SYNJ2BP	2.42	1.54	1.57

Partner-to-VDAC1 recovery ratio = partner fold recovery/VDAC1 fold recovery. Control values are normalized to 1.00. Ratios are approximate and rounded to two decimal places. These descriptive ratios are uncorrected for input protein abundance and do not quantify binding affinity.

### 3.8 SYNJ2BP knockdown preserves mitochondrial morphology in atrophied muscle

We next assessed SYNJ2BP manipulation in the separate mouse hindlimb immobilization model. Local AAV delivery was associated with increased or decreased SYNJ2BP abundance in the corresponding intervention groups (Figure 8A). Relative to immobilization alone, overexpression was accompanied by higher IP3R1, GRP75, and VDAC1 abundance, whereas knockdown was accompanied by lower abundance. Electron microscopy showed greater mitochondrial swelling, cristae disruption, and fewer mitochondrial profiles in the overexpression group. The knockdown group showed better-preserved cristae and more mitochondrial profiles than the Atrophy group (Figure 8B). Knockdown was also accompanied by higher NDUFAB1, PDHA1, MFN2, and DRP1 abundance and lower total cytochrome c and cleaved caspase-3 abundance (Figure 8C). These findings extend the association to immobilized hindlimb muscle. They do not directly measure mitochondrial calcium transfer or establish that the pathway is identical to that in supraspinatus muscle.

**FIGURE 8:**
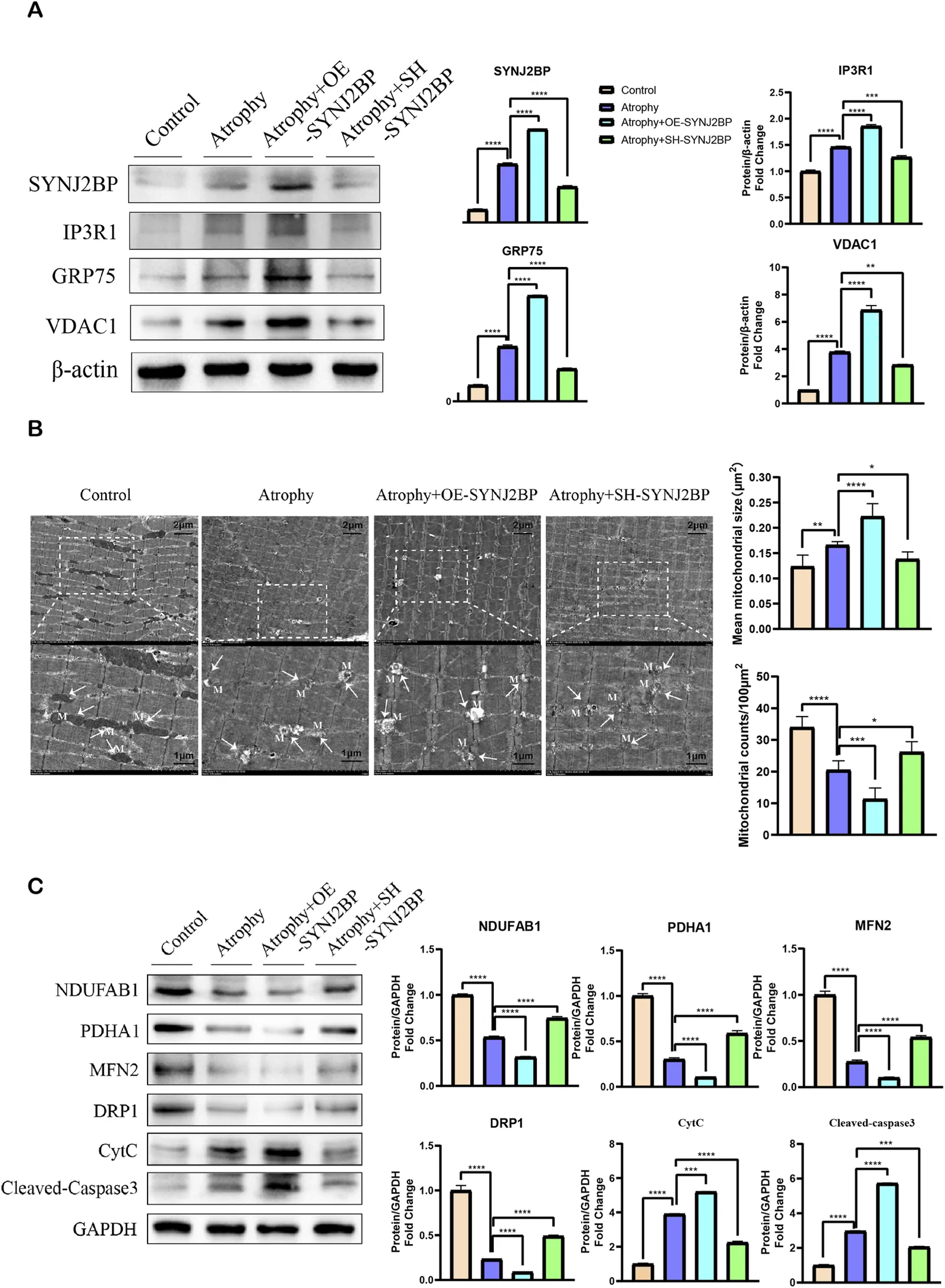
Effects of SYNJ2BP manipulation on mitochondria in immobilized muscle. Note. Control, Atrophy, Atrophy + OE-SYNJ2BP, and Atrophy + SH-SYNJ2BP mice were compared. **(A)** Western blots and quantification of SYNJ2BP, IP3R1, GRP75, and VDAC1. **(B)** Transmission electron micrographs and quantification of mitochondrial profile size and number. Scale bars: 2 μm in the upper row and 1 μm in the lower row. **(C)** Western blots and quantification of NDUFAB1, PDHA1, MFN2, DRP1, cytochrome c, and cleaved caspase-3. Data are presented as mean ± SD where quantified. *p < 0.05, **p < 0.01, ***p < 0.001, ****p < 0.0001; ns, not significant, where shown. Comparisons are indicated in the panels. Six mice were allocated to each group. Mitochondrial counts refer to profiles in sampled sections.

### 3.9 SYNJ2BP knockdown attenuates immobilization induced muscle wasting

Gross examination showed smaller gastrocnemius and tibialis anterior muscles after immobilization, with a further reduction following SYNJ2BP overexpression. In contrast, muscles from the SYNJ2BP-knockdown group were larger than those from the Atrophy group (Figure 9A). H&E staining showed corresponding changes in gastrocnemius fiber diameter: overexpression exacerbated the reduction associated with immobilization, whereas knockdown attenuated it (Figure 9B). Western blotting showed lower MYOD, MYOG, and MYHC expression and higher Atrogin-1 and MuRF-1 expression in the overexpression group than in the Atrophy group. Knockdown produced the opposite pattern (Figure 9C). Thus, the morphological and molecular findings consistently supported reduced muscle wasting after local SYNJ2BP knockdown.

**FIGURE 9:**
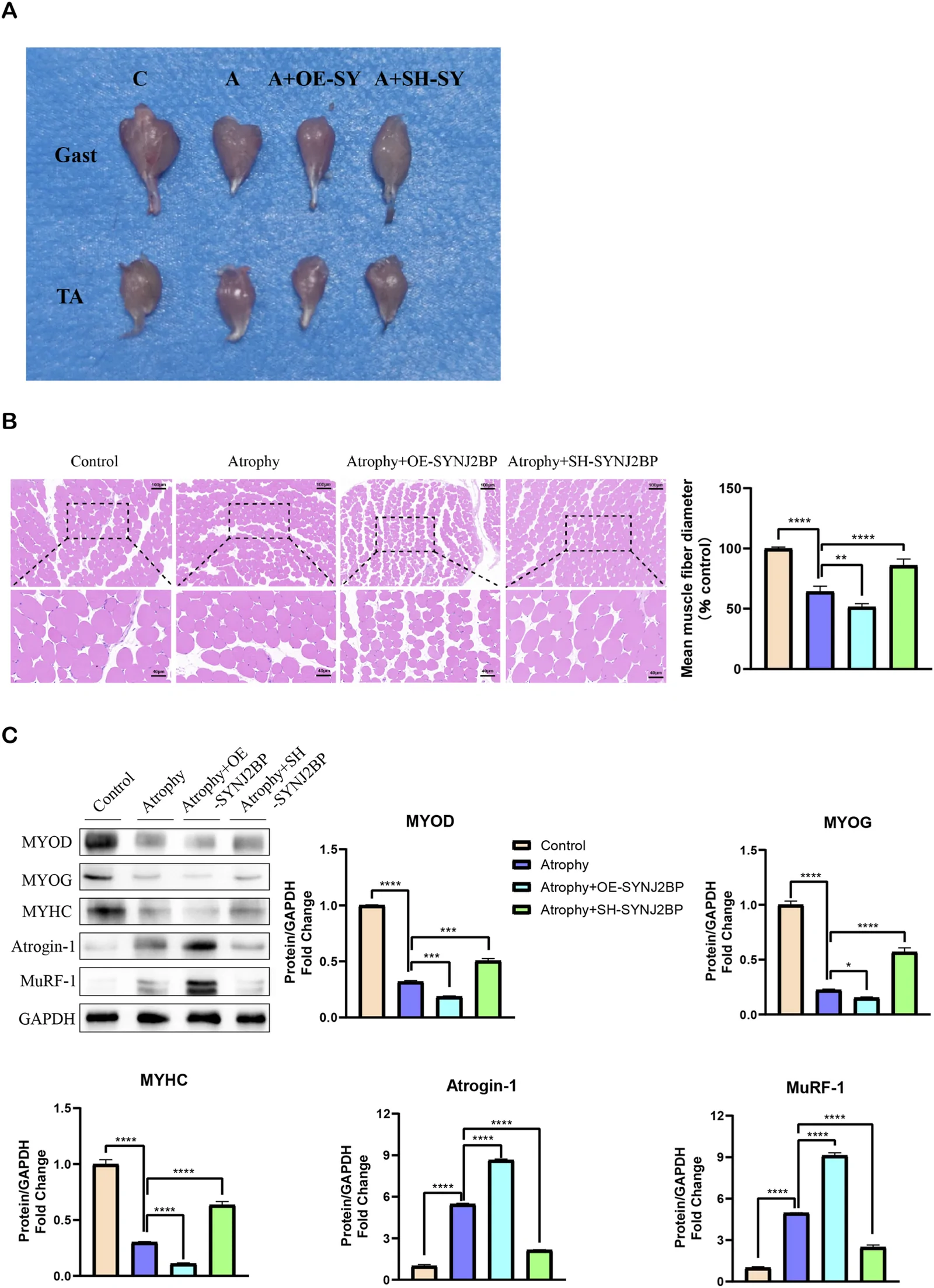
SYNJ2BP knockdown attenuates immobilization induced muscle atrophy. Note. **(A)** Representative gastrocnemius and tibialis anterior muscles from the 4 mouse groups. **(B)** H&E-stained gastrocnemius sections and muscle fiber diameter. Scale bars: 100 μm in the upper row and 40 μm in the lower row. **(C)** Western blots and quantification of MYOD, MYOG, MYHC, Atrogin-1, and MuRF-1 in gastrocnemius muscle. Data are presented as mean ± SD where quantified. *p < 0.05, **p < 0.01, ***p < 0.001, ****p < 0.0001; ns, not significant, where shown. Comparisons are indicated in the panels. Six mice were allocated to each group. Muscle fibers sampled within the same mouse are subsamples, not independent animals.

### 3.10 SYNJ2BP knockdown improves muscle mass and physical performance

Body weight was monitored in the same mice from baseline through week 6. Mean body weight was lower in the Atrophy group than in the Control group from weeks 3–6, with a further decrease in the overexpression group and an increase in the knockdown group relative to Atrophy (Figure 10A). Endpoint gastrocnemius and tibialis anterior mass showed a similar pattern (Figures 10B,C). Grip force, maximum treadmill running time, and maximum running distance were lower with immobilization and overexpression and higher in the knockdown group (Figures 10D–F). These observations were consistent with the morphological findings.

**FIGURE 10:**
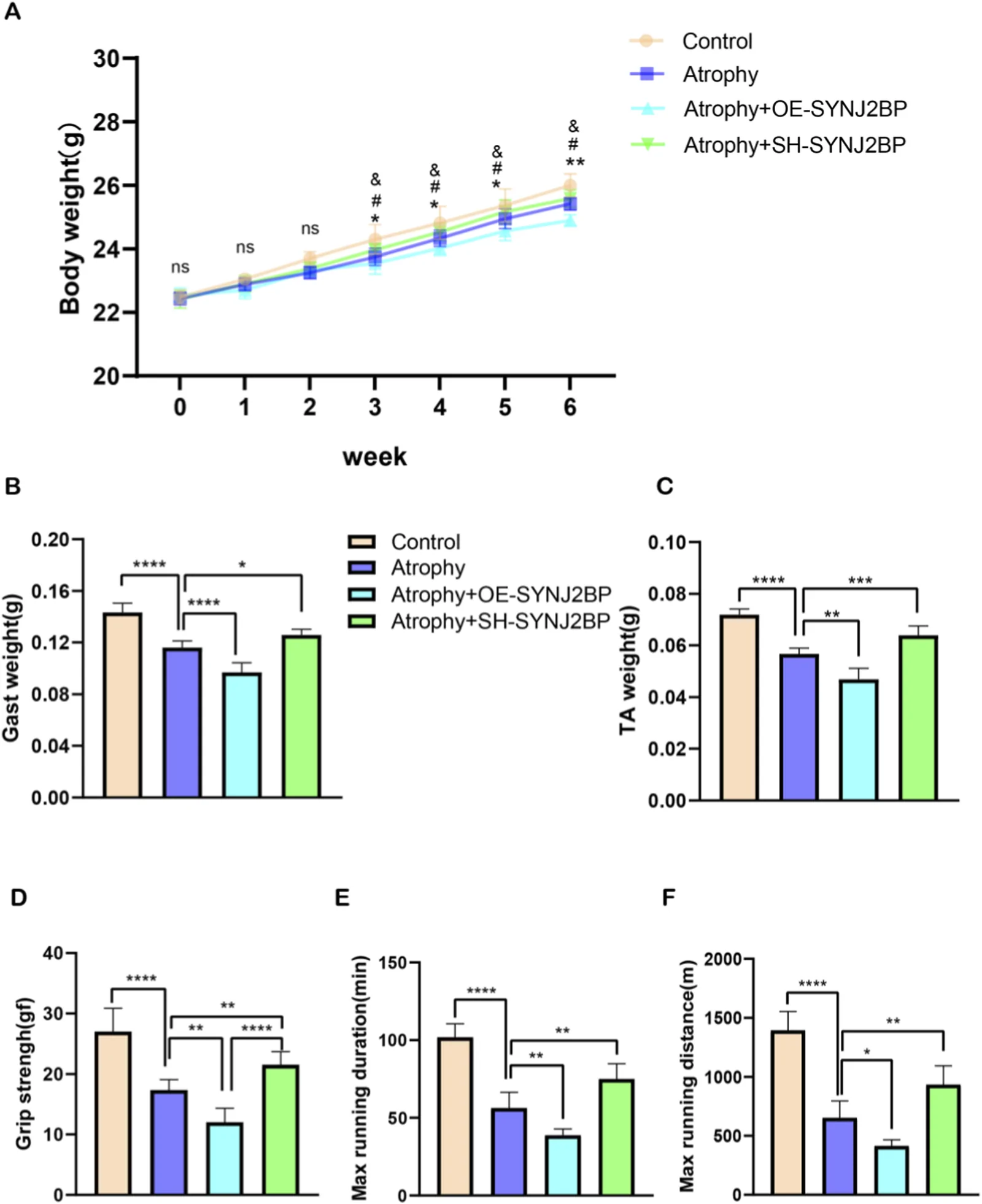
SYNJ2BP knockdown improves muscle mass and physical performance. Note. **(A)** Body weight at baseline and weeks 1–6. In panel A, * denotes Atrophy versus Control, # denotes Atrophy versus Atrophy + OE-SYNJ2BP, and & denotes Atrophy versus Atrophy + SH-SYNJ2BP. **(B,C)** Gastrocnemius **(B)** and tibialis anterior **(C)** mass. **(D)** Left hindlimb grip strength. **(E,F)** Maximum treadmill running time **(E)** and distance **(F)**. Data are presented as mean ± SD where quantified. *p < 0.05, **p < 0.01, ***p < 0.001, ****p < 0.0001; ns, not significant, where shown. Comparisons are indicated in the panels. Six mice were allocated to each group. Panel A shows repeated measurements in the same mice.

## 4 Discussion

This study identifies SYNJ2BP as a candidate regulator of skeletal muscle atrophy across human tissue, cultured cells, and mouse models. Increased SYNJ2BP abundance in atrophic human supraspinatus muscle was accompanied by changes in mitochondrial and calcium-related protein sets. In C2C12 cells, SYNJ2BP overexpression was associated with mitochondrial abnormalities, increased calcium-associated fluorescence, and altered myogenic and atrophy markers. In mice, local knockdown attenuated immobilization-induced muscle wasting and improved physical performance. These results extend evidence linking mitochondrial dysfunction to muscle atrophy during inactivity, glucocorticoid exposure, and other metabolic stresses (Chen et al., 2023; Deane et al., 2024; Hepple, 2014; Hyatt et al., 2019; Lei et al., 2024; Li et al., 2022; Liu et al., 2016; Yoo et al., 2024). They also place SYNJ2BP within the broader effort to identify molecular targets for preserving muscle function (Bonaldo and Sandri, 2013; Huang et al., 2023; Yin et al., 2021).

The results motivate a working hypothesis involving SYNJ2BP, mitochondrial abnormalities, and IP3R1/GRP75/VDAC1-associated calcium regulation (Figure 11). Increased abundance of the three proteins does not establish increased calcium transport. Likewise, greater recovery in VDAC1 immunoprecipitates is affected by bait recovery and input abundance. GRP75 recovery relative to VDAC1 was close to the control value (Table 2), which limits evidence for enhanced assembly of the entire complex. Rhod-2 fluorescence provides an additional observation but does not directly measure ER-to-mitochondria calcium flux. Work in other experimental systems provides a precedent for investigating this pathway (Meng et al., 2023), rather than direct evidence for the proposed causal sequence in skeletal muscle.

**FIGURE 11:**
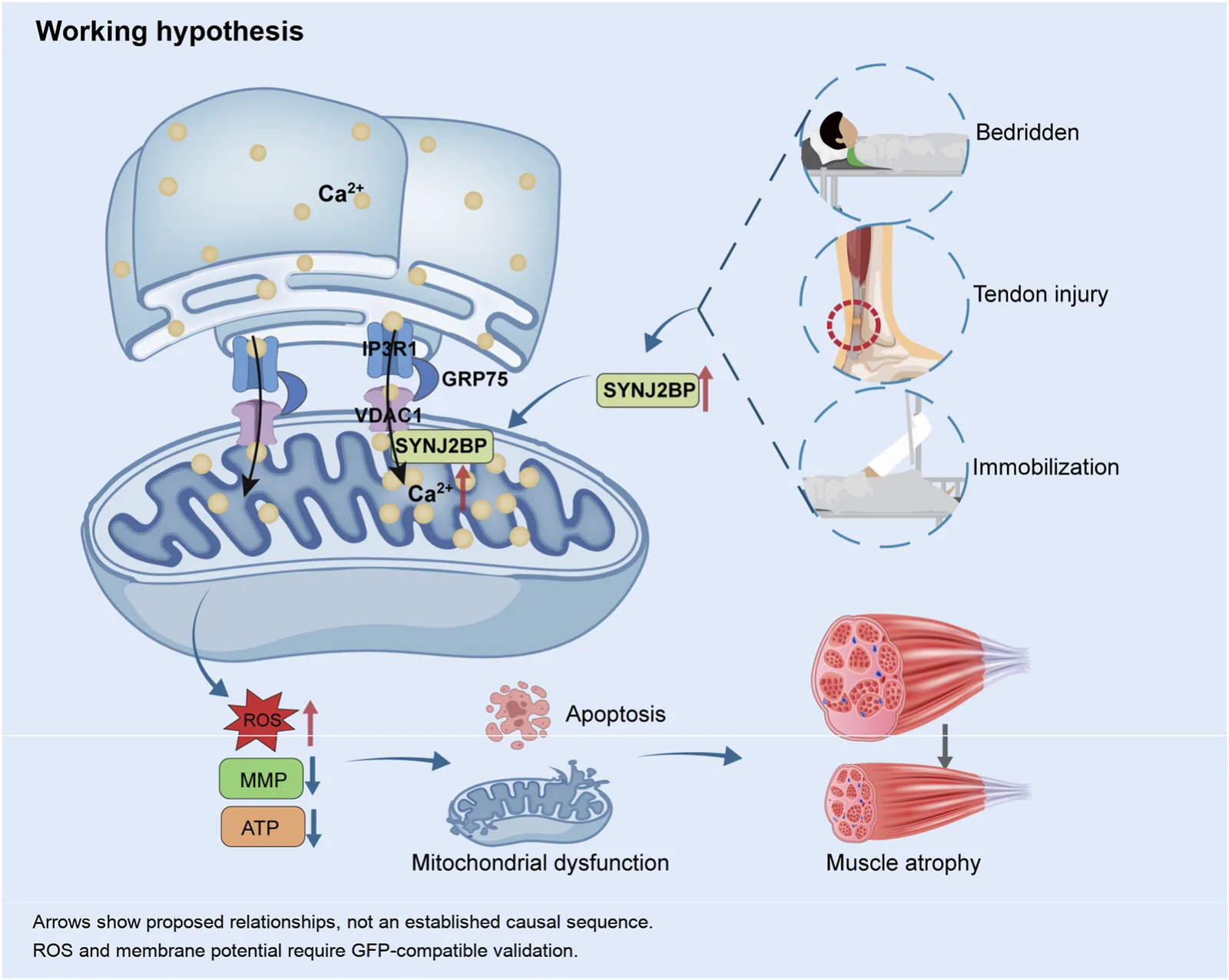
Proposed link between SYNJ2BP and skeletal muscle atrophy. Note. Working hypothesis linking SYNJ2BP, IP3R1/GRP75/VDAC1-associated calcium regulation, mitochondrial abnormalities, and muscle wasting. Arrows indicate proposed relationships. The effects on ROS, mitochondrial membrane potential, and compartment-specific calcium accumulation remain to be established. Bed rest illustrates a clinical context of muscle disuse. ER, endoplasmic reticulum; MMP, mitochondrial membrane potential; ROS, reactive oxygen species.

Published findings support a context-dependent role for SYNJ2BP. Hung et al. (2017) found that overexpression increased contacts between mitochondria and rough ER, which also illustrates why excessive expression can alter organelle organization. Pourshafie et al. (2022) observed elevated endogenous SYNJ2BP in disease-associated motor neurons and reported improved mitochondrial oxidative function after reducing SYNJ2BP. In contrast, Song et al. (2024) reported protective effects of SYNJ2BP in nucleus pulposus cells through ER–mitochondria contacts and mitochondrial zinc homeostasis. Together, these studies indicate that SYNJ2BP function depends on tissue context and expression level. Supraphysiological expression may induce nonspecific cellular stress, which remains a possible contributor to the phenotypes observed in our overexpression models.

VBIT-4 was originally developed as a VDAC1-interacting compound that inhibited oligomerization and apoptosis in the tested systems (Ben-Hail et al., 2016). Belosludtsev et al. (2023) reported that 5 μM VBIT-4 reduced oxidative injury in endothelial cells exposed to high glucose for 36 h and separately examined genetic reduction of VDAC1 in fibroblasts. These findings support investigation of VDAC-related processes, although pharmacological effects may differ between cell types. An eLife reviewed preprint also reports VDAC1-independent membrane effects of VBIT-4 under its test conditions (Ravishankar et al., 2026). In our experiments, VBIT-4 reduced SYNJ2BP abundance as well as several downstream readouts. The observed attenuation therefore cannot be assigned exclusively to VDAC1 inhibition or selective disruption of the IP3R1/GRP75/VDAC1 complex. Independent genetic perturbation of a complex component, accompanied by target and SYNJ2BP expression measurements, would help distinguish these explanations.

The tendon-transection and hindlimb-immobilization models provide complementary evidence. The first links SYNJ2BP expression to supraspinatus injury, whereas the second tests local manipulation during reduced hindlimb use and permits assessment of muscle mass and performance. These settings differ in anatomical site, age at entry, injury-associated inflammation, and mechanical loading. Directionally consistent findings therefore support broader relevance of SYNJ2BP but do not prove a common mechanism. Moreover, saline served as the comparator for the AAV groups. Without matched empty-vector and non-targeting shRNA controls, vector-related effects and sequence-specific off-target effects cannot be fully separated from SYNJ2BP manipulation. The findings do not yet establish efficacy in human rotator cuff disease, aging, or systemic illness.

Several limitations affect interpretation. The human proteomic cohort was small and observational. Changes in myogenic markers do not by themselves establish mature-myotube atrophy. Vector-encoded GFP may interfere with green-channel fluorescence, limiting interpretation of the ROS and membrane-potential assays. Rhod-2 fluorescence is sensitive to probe loading and localization and does not resolve calcium-transfer kinetics. Co-immunoprecipitation is influenced by bait recovery and input abundance, whereas total cytochrome c abundance does not demonstrate cytosolic release. Supraphysiological SYNJ2BP expression may also cause nonspecific cellular stress. Independent genetic perturbation of VDAC1, GRP75, or IP3R1 and direct measurements of compartment-specific calcium dynamics would help establish whether this pathway mediates the observed phenotypes. The body-weight trajectories are interpreted descriptively because repeated measurements within animals limit inference from individual time-point comparisons.

## 5 Conclusion

The combined observations implicate SYNJ2BP in mitochondrial abnormalities and skeletal muscle wasting. SYNJ2BP abundance was higher in atrophic supraspinatus muscle, and its manipulation was associated with altered mitochondrial morphology, muscle-related markers, and functional outcomes in experimental models. The calcium-associated fluorescence and co-immunoprecipitation findings motivate investigation of IP3R1/GRP75/VDAC1-associated regulation, but they do not establish enhanced complex assembly or a complete causal pathway. Local SYNJ2BP reduction may help preserve muscle structure and function, while its physiological relevance and the underlying calcium-signaling mechanism warrant further investigation.

## Data Availability

The original contributions presented in the study are included in the article/supplementary material, further inquiries can be directed to the corresponding authors.
